# Morphology and phylogeny identify two new species and one new subspecies of *Podoscypha* from Yunnan Province, Southwest China

**DOI:** 10.3389/fmicb.2023.1151365

**Published:** 2023-02-28

**Authors:** Jing Si, Yi-Zhe Zhang, Jia-Qi Liang, Hai-Jiao Li

**Affiliations:** ^1^Institute of Microbiology, School of Ecology and Nature Conservation, Beijing Forestry University, Beijing, China; ^2^National Institute of Occupational Health and Poison Control, Chinese Center for Disease Control and Prevention, Beijing, China

**Keywords:** macrofungi, species diversity, Podoscyphaceae, taxonomy, phylogeny, fungal resources

## Abstract

In this study, *Podoscypha* was taxonomically and phylogenetically evaluated. In total, five specimens collected from the tropical areas of Yunnan Province in Southwest China were studied. In combination with morphological observations and phylogenetic analyses based on ITS and LSU loci, two new species and one new subspecies, *Podoscypha subinvoluta*, *P. tropica*, and *P. petalodes* subsp. *cystidiata*, respectively, were discovered. The illustrated descriptions of the new species and subspecies are provided. Moreover, the main morphological differences between related species are discussed.

## 1. Introduction

*Podoscypha* Pat. belongs to Podoscyphaceae D.A. Reid, Polyporales Gäum., and approximately 50 species have been accepted in the genus worldwide (Patouillard, 1900; Boidin, 1959, 1960; Reid, 1965; Dhingra, 1987; Ryvarden, 1997, 2015, 2020; Douanla-Meli and Langer, 2004; Drechsler-Santos et al., 2007; Bernicchia and Gorjón, 2010; Wu et al., 2019). The genus is characterized by flabelliform to infundibuliform, stipitate to shortly stipitate or sessile basidiocarps, hymenophore smooth to more or less rugose, a dimitic hyphal structure with clamped generative hyphae, thin-to thick-walled cystidia and hyaline, thin-walled, smooth, ellipsoid to cylindrical, acyanophilous, and negative in Melzer’s reagent basidiospores (Bernicchia and Gorjón, 2010; Wu et al., 2019).

Phylogenetic studies have revealed that *Podoscypha* belongs to Podoscyphaceae, Polyporales (Larsson, 2007; Sjökvist et al., 2012; Binder et al., 2013; Justo et al., 2017; Wu et al., 2019). The approximately 12 *Podoscypha* species examined showed that *Podoscypha* belonged to the residual polyporoid clade and appeared to group with *Abortiporus biennis* (Bull.) Singer (Binder et al., 2013; Justo et al., 2017; Wu et al., 2019). However, no sequences of the type species *P. nitidula* (Berk.) Pat. were examined. Further studies are needed for constructing a natural phylogeny of *Podoscypha* with other related genera.

By the end of 2022, only four *Podoscypha* species: *Podoscypha brasiliensis* D.A. Reid, *P. elegans* (G. Mey.) Pat., *P. simulans* (D.A. Reid) Sheng H. Wu, and *P. yunnanensis* C.L. Zhao had been discovered in China (Wu, 2003; Dai, 2011; Wu et al., 2019). During field investigations on macrofungi diversity in the tropical areas in Yunnan Province, China, several collections of *Podoscypha* were obtained. Morphological and phylogenetic analyses showed that these represented two new species and one new subspecies. In this study, the species are described with illustrated morphological description, phylogeny, and comparisons with morphologically similar or phylogenetically related species.

## 2. Materials and methods

### 2.1. Morphology

All the studied specimens were processed and deposited in the National Institute of Occupational Health and Poison Control, Chinese Center for Disease Control (NIOHP, China CDC). Macromorphological descriptions were based on the field notes and color photographs of basidiocarps. Color codes were matched based on Petersen (1996). The microscopic procedure was performed according to Reid (1965), Douanla-Meli and Langer (2004), and Wu et al. (2019). Sections were studied at a magnification of ×1,000 with a Nikon E 80i microscope and phase-contrast illumination. Line drawings were made with the aid of a drawing tube. When describing basidiospores, the abbreviation n/m/p means that “n” basidiospores from “m” basidiomata of “p” collections were measured. The dimensions for basidiospores are given using the following notation form (a−) b−c (−d). The range “b−c” contains a minimum of 90% of the measured values, and extreme values (a, d) are given in parentheses. L means the spore length (arithmetic average of all spores). W means the spore width (arithmetic average of all spores). Q is the “length/width ratio” of a spore in side view; Q_m_ means the average Q of all basidiospores measured ± sample standard deviation. In the text, the following abbreviations were used: IKI = Melzer’s reagent, IKI− = both in amyloid and non-dextrinoid, KOH = 5% potassium hydroxide, CB = cotton blue, CB+ = cyanophilous, and CB− = acyanophilous.

### 2.2. DNA extraction, PCR amplification, and phylogenetic analysis

The Phire® Plant Direct PCR Kit (Finnzymes Oy, Finland) was used to obtain PCR products from dried specimens, according to the manufacturer’s instructions with some modifications (Li et al., 2015). ITS5/ITS4 were used to amplify the internal transcribed spacer (ITS) regions (White et al., 1990). LR0R/LR5 (Vilgalys and Hester, 1990) were used to amplify nuclear large subunit (LSU) rDNA. The PCR procedure for amplifying ITS and LSU sequences was as follows: initial denaturation at 98°C for 5 min; 35 cycles at 98°C for 5 s, 58°C and 52°C for 5 s, and 72°C for 5 s; and a final extension at 72°C for 10 min, respectively. The PCR products were qualified by electrophoresis and sequenced at Sangon Biotech, China, with the same primers. All newly generated sequences were first aligned and then deposited in GenBank. The results indicated that they were all *Podoscypha* species.

In addition to the sequences obtained from this study, the sequences of other taxa in *Podoscypha* were downloaded from GenBank, and *Abortiporus biennis* was selected as the outgroup for phylogenetic analysis, mainly based on the reports of Binder et al. (2013) and Wu et al. (2019). The detailed information is shown in Table 1. The sequences were aligned using ClustalX 1.83 (Chenna et al., 2003) and optimized manually in BioEdit 7.0.5.3 (Hall, 1999) prior to the phylogenetic analysis.

**Table 1:** Taxa information and GenBank accession numbers of the sequences used in this study.

Species	Specimen No.	Locality	ITS	nLSU	Literature
*Abortiporus biennis*	FD 319	Massachusetts, United States	KP135300	KP135195	Binder et al. (2013)
*Podoscypha bolleana*	32034	−	JQ675334	−	Binder et al. (2013)
*P. bolleana*	32032	−	JQ675332	−	Binder et al. (2013)
*P. bolleana*	CBS 333.66	Central African Republic	JN649354	JN649354	Sjökvist et al. (2012)
*P. brasiliensis*	17586	−	JQ675312	−	Binder et al. (2013)
*P. brasiliensis*	GXU 2169	China	−	MG356489	−
*P. brasiliensis*	LR 37812	Venezuela	JN649355	JN649355	Sjökvist et al. (2012)
*P. bubalina*	17500	−	JQ675311	−	Binder et al. (2013)
*P. cristata*	8667	−	JQ675320	−	Binder et al. (2013)
*P. disseminata*	DMC 232	−	JQ675326	−	Binder et al. (2013)
*P. elegans*	CBS 332.66	Pakistan	−	MH870450	Vu et al. (2019)
*P. elegans*	CBS 426.51	Argentina	JN649356	JN649356	Sjökvist et al. (2012)
*P. elegans*	CBS 426.51	Argentina	−	MH868453	Vu et al. (2019)
*P. fulvonitens*	17483	−	JQ675315	−	Binder et al. (2013)
*P. fulvonitens*	18332	−	JQ675316	−	Binder et al. (2013)
*P. fulvonitens*	C 1	−	JQ675322	−	Binder et al. (2013)
*P. gillesii*	32036	−	JQ675335	−	Binder et al. (2013)
*P. gillesii*	GXU 2176	Guangxi, China	−	MG356793	−
*P. involuta*	CBS 113.74	Central African Republic	JN649358	JN649358	Sjökvist et al. (2012)
*P. involuta*	CBS 654.84	Gabon	MH861804	MH873497	Sjökvist et al. (2012)
*P. mellissii*	LR 41658	Jamaica	JN649359	JN649359	Sjökvist et al. (2012)
*P. moelleri*	17588	−	JQ675313	−	Binder et al. (2013)
*P. multizonata*	3005	Germany	JN710581	JN710581	Miettinen et al., 2012
*P. multizonata*	CBS 662.84	France	MH861808	−	Sjökvist et al. (2012)
*P. multizonata*	CBS 663.84	France	MH861809	MH873501	Sjökvist et al. (2012)
*P. multizonata*	CBS 661.84	France	MH861807	MH873500	Sjökvist et al. (2012)
*P. multizonata*	CBS 660.84	France	MH861806	MH873499	Sjökvist et al. (2012)
*P. multizonata*	Jahn 751012	Germany	JN649360	JN649360	Sjökvist et al. (2012)
*P. parvula*	CBS 331.66	Central African Republic	JN649361	JN649361	Sjökvist et al. (2012)
*P. parvula*	DAOM171399	−	−	AF261534	Moncalvo et al. (2002)
*P. parvula*	DMC 226	−	JQ675328	−	Binder et al. (2013)
*P. parvula*	FP-150577	−	JQ675338	−	Binder et al. (2013)
***P. petalodes* subsp. *cystidiata***	**140721–14**	**Yunnan, China**	**OQ305832**	**OQ305827**	**Present study**
***P. petalodes* subsp. *cystidiata***	**140721–15 (Holotype)**	**Yunnan, China**	**OQ305833**	**OQ305828**	**Present study**
*P. petalodes* subsp. *rosulata*	AFTOL-ID 1931	Pakistan	−	EF537892	−
*P. petalodes* subsp. *rosulata*	CBS 332.66	Pakistan	JN649363	JN649363	Sjökvist et al. (2012)
*P. petalodes* subsp. *rosulata*	CBS 659.84	Pakistan	JN649362	JN649362	Sjökvist et al. (2012)
*P. petalodes* subsp. *rosulata*	CBS 659.84	Pakistan	−	MH873498	Sjökvist et al. (2012)
*P. petalodes*	DSH 98–001	−	−	AF518639	Hibbett and Binder (2002)
*P. ravenelii*	CBS 664.84	United States	JN649364	JN649364	Sjökvist et al. (2012)
***P. subinvoluta***	**140721–10 (Holotype)**	**Yunnan, China**	**OQ305836**	**OQ305831**	**Present study**
*P. subinvoluta*	E. Larsson	Thailand	JN649357	−	Sjökvist et al. (2012)
***P. tropica***	**140719–19 (Holotype)**	**Yunnan, China**	**OQ305834**	**OQ305830**	**Present study**
***P. tropica***	**20190729–69**	**Yunnan, China**	**OQ305835**	**OQ305829**	**Present study**
*P. venustula*	CBS 656.84	French Guiana	JN649367	JN649367	Sjökvist et al. (2012)
*P. venustula*	Cui 16923	Puerto Rico	−	ON417231	−
*P. venustula*	LR 40821	Venezuela	JX109851	JX109851	Sjökvist et al. (2012)
*P. venustula*	LR 40821	Venezuela	−	JN649366	Sjökvist et al. (2012)
*P. vespillonea*	CBS 111.74	−	JN649368	JN649368	Sjökvist et al. (2012)
*P. vespillonea*	CBS 111.74	−	MH872572	MH872572	Sjökvist et al. (2012)
*P. vespillonea*	CBS 348.66	−	MH858820	MH870457	Sjökvist et al. (2012)
*P. yunnanensis*	CLZhao 3963	Yunnan, China	MK298400	MK298404	Wu et al. (2019)
*P. yunnanensis*	CLZhao 4035	Yunnan, China	MK298403	MK298407	Wu et al. (2019)
*P. yunnanensis*	CLZhao 3973	Yunnan, China	MK298401	MK298405	Wu et al. (2019)
*P. yunnanensis*	CLZhao 3979	Yunnan, China	MK298402	MK298406	Wu et al. (2019)
*Podoscypha* sp.	DIS 296f	Cameroon	−	DQ327658	Crozier et al. (2006)
*Podoscypha* sp.	LR43794	Costa Rica	−	JN649365	Sjökvist et al. (2012)
*Podoscypha* sp.	MKS3a	India	−	OP684268	−
*Podoscypha* sp.	SCHD9	West Bengal, India	−	KY594043	−
*Podoscypha* sp.	TYY2020-55	China	−	OM281038	−

New species are in bold.

Maximum parsimony (MP) bootstrap analysis was performed using PAUP* 4.0b10 (Swofford, 2002); Bayesian inference (BI) analysis was performed using MrBayes 3.1.2 (Ronquist and Huelsenbeck, 2003); maximum likelihood (ML) analysis was performed using RAxML 7.2.6 (Stamatakis, 2006) for the phylogenetic analysis of the aligned datasets.

For analyzing BI, the best-fit models of nucleotide substitution were selected by the hierarchical likelihood ratio tests (hLRT; Huelsenbeck and Crandall, 1997; Posada and Crandall, 2001) using MrModelTest 2.2 (Posada and Crandall, 1998; Nylander, 2004). The best-fit models were GTR + G for ITS and LSU. Four Markov chains were set to run for 2 and 4 million generations for the ITS and LSU datasets, respectively, and then, automatically terminated using the stoprul and stopval commands when the standard deviation of the split frequencies fell below 0.01, with sampling for every 100th generation. The first 25% of trees were discarded (Ronquist and Huelsenbeck, 2003). Phylogenetic trees were visualized using TreeView (Page, 1996). Branches that received bootstrap supports for MP (≥75%), BPP (≥0.95), and ML (≥75%) were considered significantly supported.

## 3. Results

### 3.1. Phylogenetic analyses

**Figure 1:**
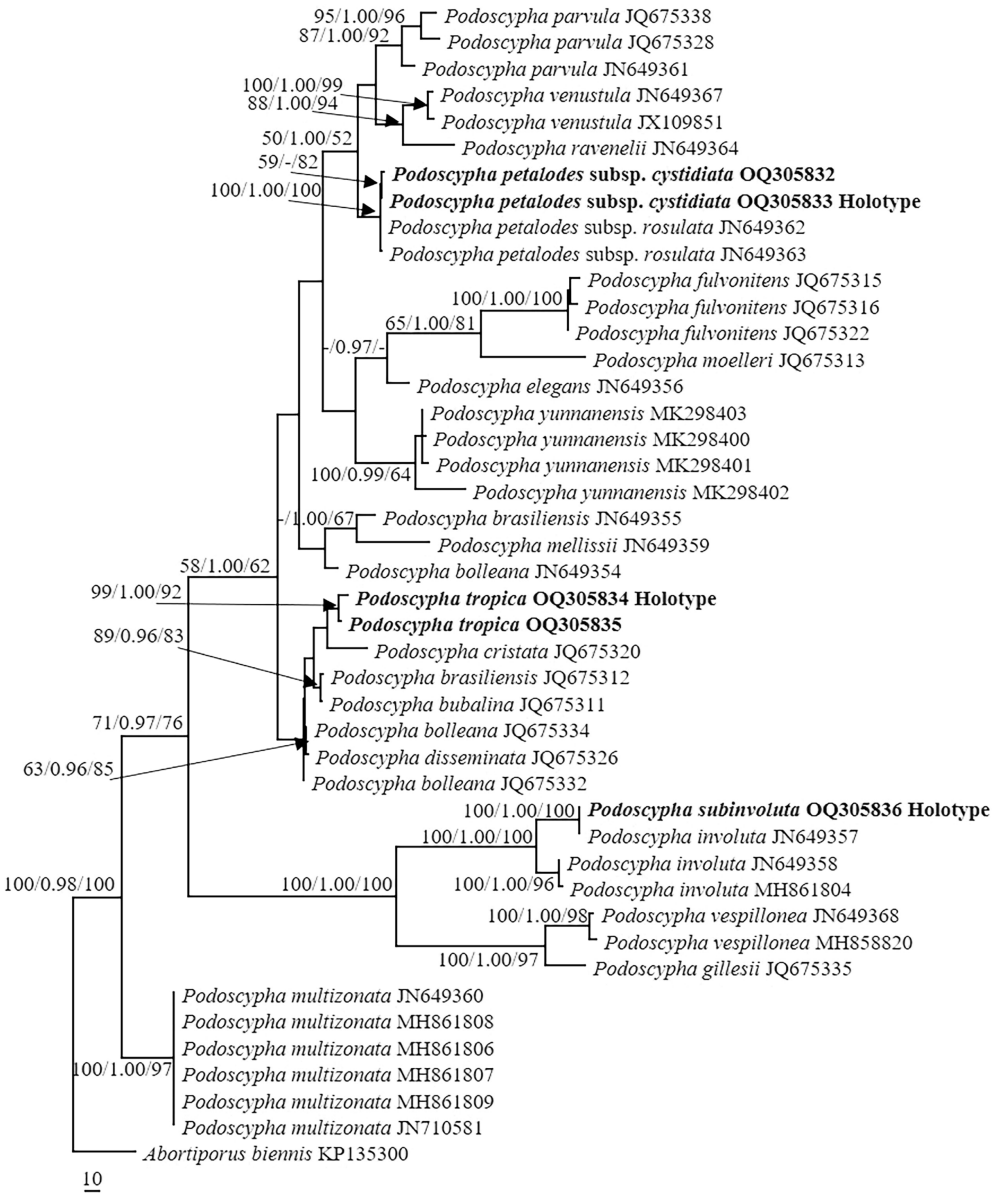
Phylogeny of *Podoscypha* by maximum parsimony (MP) analyses based on internal transcribed spacer (ITS) sequences. Branches are labeled with MP bootstrap >50%, Bayesian posterior probabilities >0.95, and maximum likelihood bootstrap >50%. New species and subspecies are indicated in bold.

The ITS dataset consisted of 44 sequences representing 22 taxa, of which 21 were *Podoscypha*. The aligned length of the ITS sequence was 835 bp, which contained 447 parsimony informative sites. MP analysis revealed two equally parsimonious trees (tree length = 1,541, CI = 0.635, RI = 0.816, RC = 0.518, and HI = 0.316). BI and ML analyses showed almost identical tree topologies as MP analysis, with an average standard deviation of split frequencies of 0.007834 for BI analysis. Additionally, the MP tree is presented with the support values from MP, BI, and ML analyses (Figure 1).

**Figure 2:**
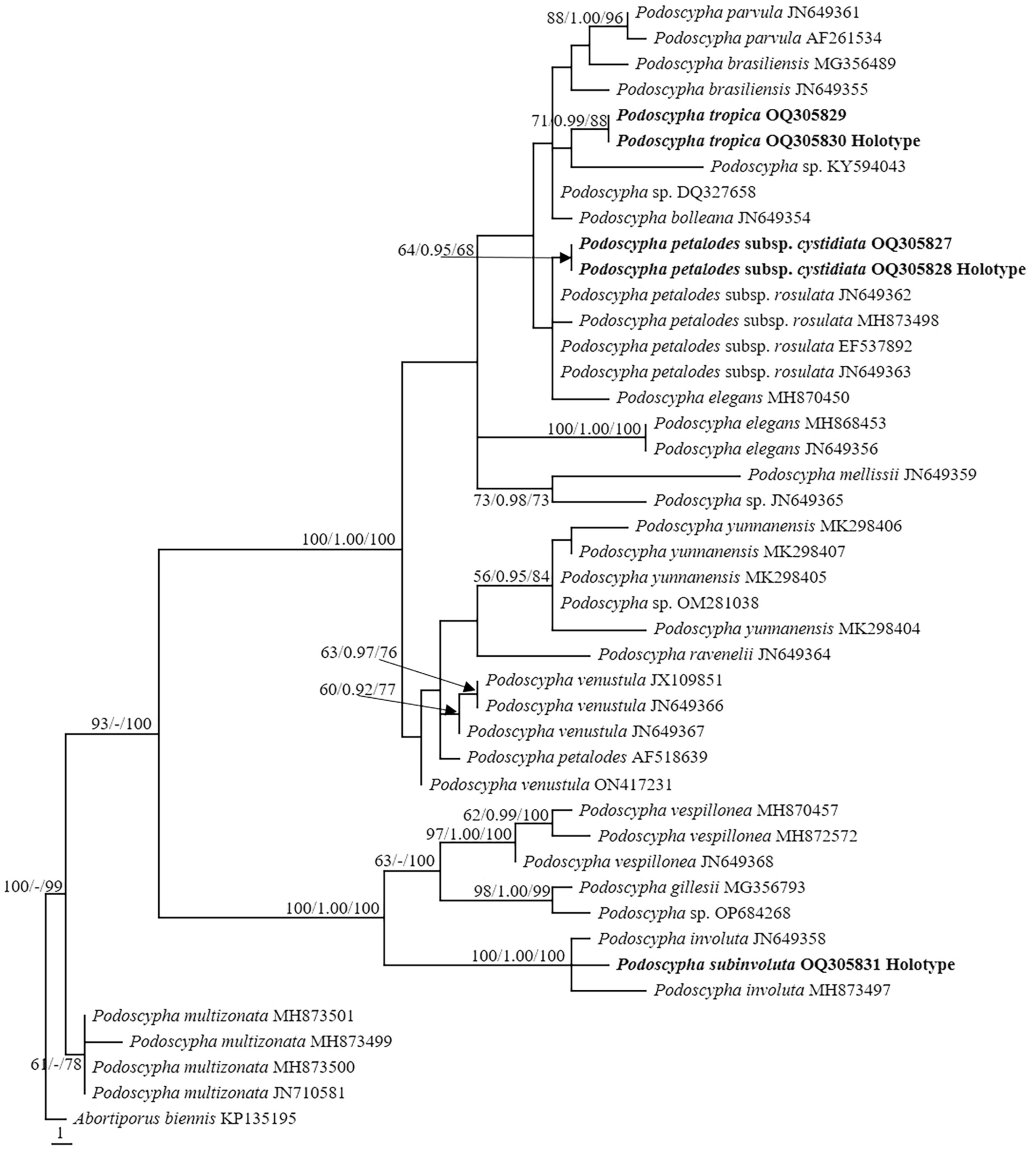
Phylogeny of *Podoscypha* by maximum parsimony (MP) analyses based on nuclear large subunit (LSU) sequences. Branches are labeled with MP bootstrap >50%, Bayesian posterior probabilities >0.95, and maximum likelihood bootstrap >50%. New species and subspecies are indicated in bold.

The LSU dataset consisted of 44 sequences, representing approximately 18 taxa, of which 17 were *Podoscypha*. The aligned length of the LSU sequence was 926 bp, which contained 72 parsimony informative sites. MP analysis revealed 44 equally parsimonious trees (tree length = 161, CI = 0.745, RI = 0.917, RC = 0.684, HI = 0.255). BI and ML analyses resulted in almost identical tree topologies to MP analysis, with an average standard deviation of split frequencies of 0.006556 for BI analysis. Additionally, the MP tree is presented with the support values from MP, BI, and ML analyses (Figure 2).

The topology of both ITS and LSU sequences was similar to the results of Wu et al. (2019). The phylogeny inferred from the ITS showed that *Podoscypha petalodes* subsp. *Cystidiata* formed a moderately supported lineage (59/−/82, Figure 1) and then clustered with *P. petalodes* subsp. *rosulata* with high support (100/1.00/100, Figure 1). LSU analysis revealed that two sequences of *P. petalodes* subsp. *cystidiata* grouped together (64/0.95/68, Figure 2) and then weakly grouped with *P. petalodes* subsp. *rosulata*. One sequence was labeled as *P. elegans* (MH870450). *Podoscypha subinvoluta* clustered with one sequence of *P. involuta* (JN649357) with high support (100/1.00/100, Figure 1), sister to the lineage of two other sequences of *P. involuta* in ITS analysis (Figure 1). In the LSU analysis, *P. subinvoluta* grouped with two sequences of *P. involuta* with high support (100/1.00/100, Figure 2). In total, two sequences of *P. tropica* clustered together with high support in both ITS and LSU analyses (ITS: 99/1.00/92, LSU: 71/0.99/88, Figures 1, 2) and then grouped with *P. cristata*, *P. brasiliensis*, *P. bubalina*, *P. bolleana*, *P. disseminata*, and *Podoscypha* sp. (KY594043, Figures 1, 2).

### 3.2. Taxonomy

**Figure 3:**
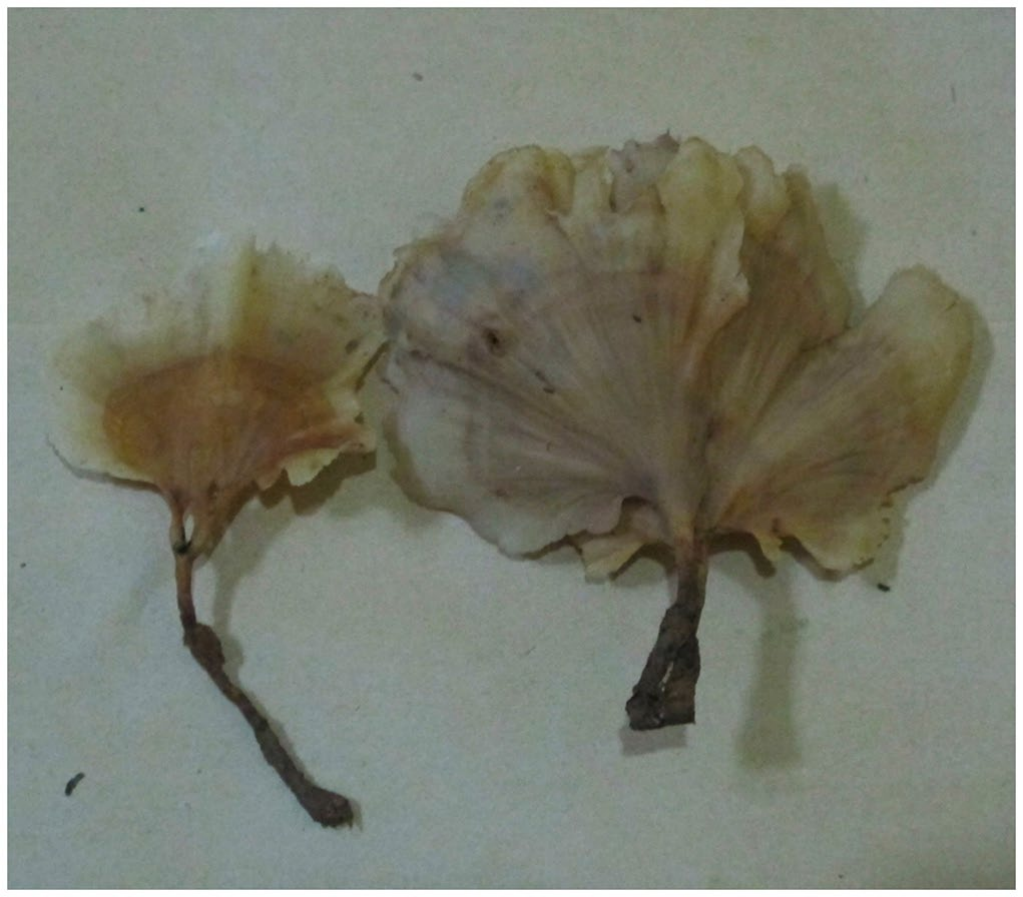
Basidiomata of *Podoscypha petalodes* subsp. *cystidiata*.

**Figure 4:**
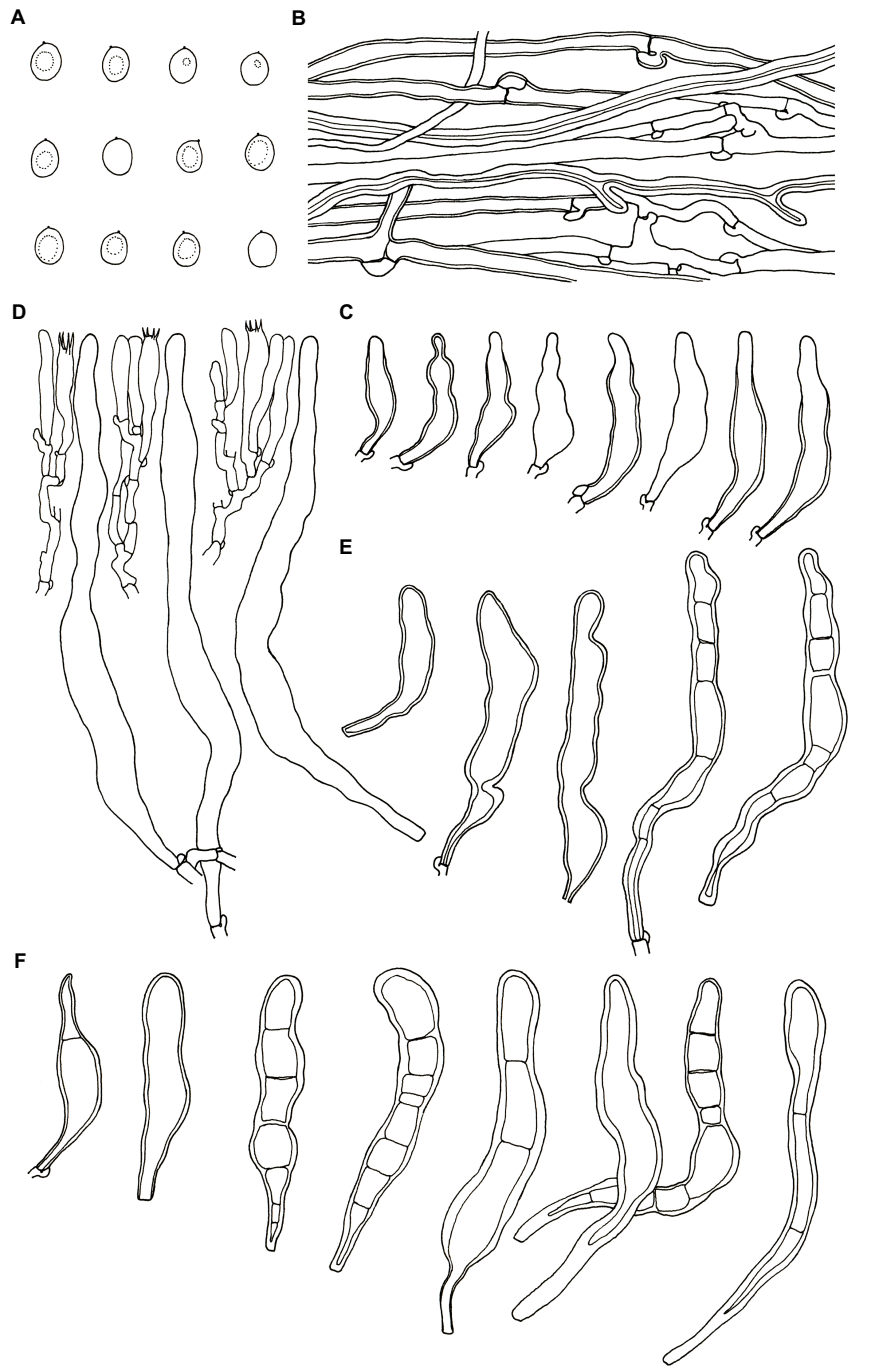
Microscopic structures of *Podoscypha petalodes* subsp. *cystidiata* (140721–15, holotype). **(A)** Basidiospores, **(B)** hyphae from context, **(C)** cystidia, **(D)** basidia, basidioles, and gloeocystidia from the hymenium, **(E)** pilocystidia, and **(F)** caulocystidia. (Bars: **(A)** = 5 μm, **(B–F)** = 10 μm). Drawn by Hai-Jiao Li.

***Podoscypha petalodes* subsp. *cystidiata*** Jing Si & Hai J. Li subsp. nov. (Figures 3, 4).

MycoBank no.: MB847438.

*Diagnosis*—*Podoscypha petalodes* subsp. *cystidiata* differs from *P. petalodes* subsp. *floriformis*, *P. petalodes* subsp. *petalodes*, and *P. petalodes* subsp. *rosulata* in having thin-to thick-walled cystidia in the hymenium.

*Holotype*—China. Yunnan Province, Xishuangbanna, Mengla County, Xishuangbanna Tropical Botanical Garden, on the base of a living angiosperm tree, 140721–15 (GenBank accession numbers: OQ305833-ITS, OQ305828-LSU).

*Etymology*—*cystidiata* referring to the presence of cystidia in the hymenium.

*Habitat and distribution*—Gregarious on angiosperm trees or fallen angiosperm branches, at present only discovered from its type locality, summer.

*Known distribution*—China (Yunnan).

*Description*—*Basidiocarps* annual, gregarious, stipitate, without odor or taste, corky when fresh and hard corky upon drying. *Pilei* spathulate to flabelliform, up to 4 cm high and 5 cm wide. *Pileal surface* glabrous, concentrically and radially zonate; cinnamon, yellowish brown, pinkish buff to clay-buff at the center and near the stipe when fresh, cinnamon, yellowish brown to orange-brown when dry, and gradually lightening toward the margin. The margin is cream to buff when fresh, pinkish buff to cinnamon-buff when dry and wavy. *Hymenophore surface* smooth, concentrically and radially zonate, cream, buff, to cinnamon-buff when fresh, flesh-pink to clay-buff when dry. *Stipe* surface glabrous, cinnamon, yellowish brown, pinkish buff to clay-buff near the cap, and dark brown to black toward the base when fresh; the color is similar to the pileal surface when dry, up to 3 cm long and 1.5–4 mm in diameter.

*Hyphal structure*—dimitic, generative hyphae with clamps, hyaline, thin-to thick-walled, branched; skeletal hyphae colorless, thick-walled with a wide to narrow lumen, unbranched to rarely branched; IKI−, CB−, tissues unchanged in KOH. Generative hyphae in the pileal context dominant, hyaline, thin-to thick-walled, branched, almost parallel along the pileal surface, 2–5 μm in diameter; skeletal hyphae in the pileal context hyaline, thick-walled, with a wide to narrow lumen, unbranched to rarely branched, 2–4 μm in diameter; generative hyphae in the stipe trama similar to that in the pileal context, 3–4 μm in diameter; skeletal hyphae in the stipe trama similar to that in the pileal context, 1.5–3.5 μm in diameter.

*Microstructure*—*Basidia* clavate to cylindrical, with four sterigmata and a basal clamp, 20–25 × 4–5 μm; basidioles dominant, in shape similar to basidia, but slightly smaller; *Gloeocystidia* abundant, thin-walled, cylindrical to subcylindrical, usually swollen near the base and with obtuse apex, traversing the entire width of the thickened hymenium, 105–155 × 10–13 μm; *Cystidia* hyaline, fusoid, thin-to thick-walled, 30–64 × 7–9 μm; *Pilocystidia* hyaline, clavate to cylindrical, thick-walled, with or without secondary septata, sometimes encrusted with irregular crystals, 45–101 × 9–13 μm; *Caulocystidia* more or less similar to pilocystidia, 58–110 × 5–13 μm; *Basidiospores* subglobose, broadly ellipsoid, ellipsoid to ovate, hyaline, thin-walled, smooth, often monoguttulate, IKI−, CB−, [60/2/2] (3.5−) 3.8–4.8 (−5) × 3–4 (−4.5) μm, L = 4.2 μm, W = 3.54 μm, Q = (1.05−) 1.08–1.34 (−1.4), and Q_m_ = 1.19 ± 0.09.

*Additional specimen examined (paratype)*—China. Yunnan Province, Xishuangbanna, Mengla County, Xishuangbanna Tropical Botanical Garden, on fallen angiosperm branches, 140721-14 (GenBank accession numbers: OQ305832-ITS, OQ305827-LSU).

**Figure 5:**
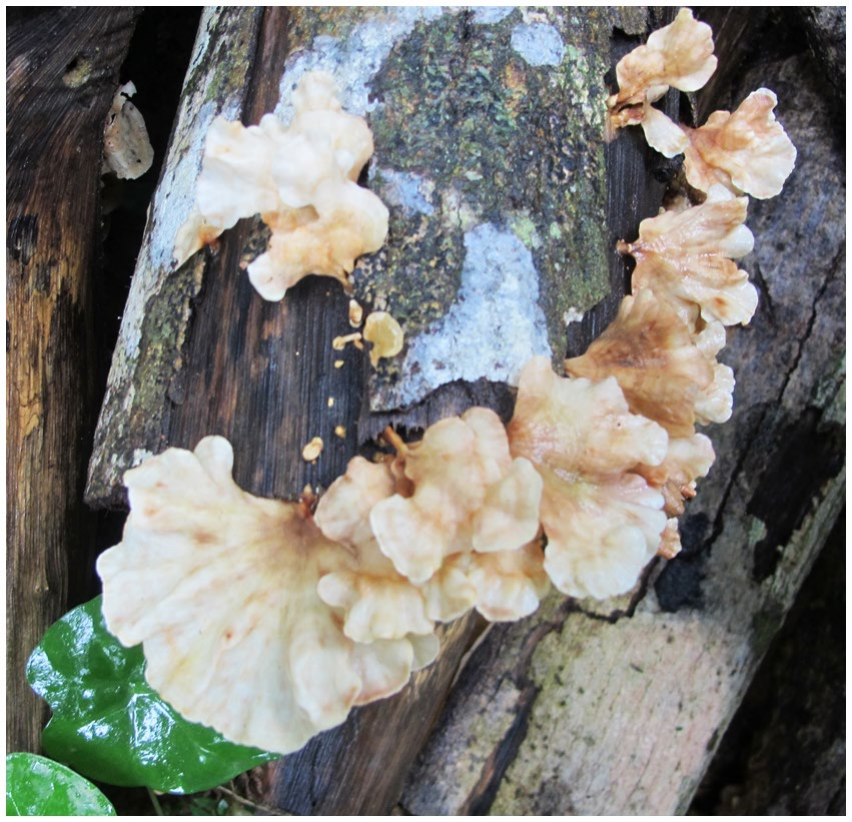
Basidiomata of *Podoscypha subinvoluta*.

**Figure 6:**
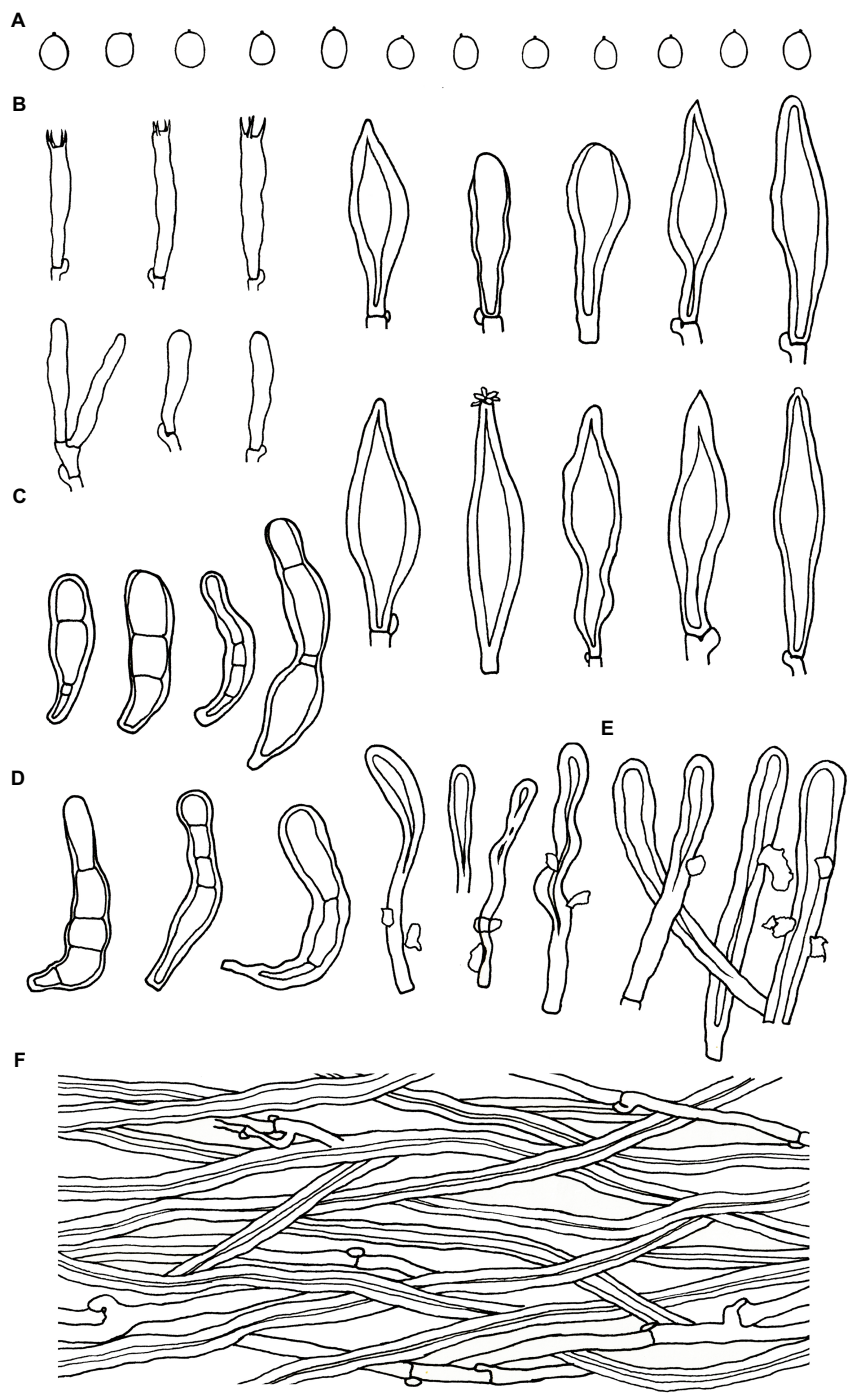
Microscopic structures of *Podoscypha subinvoluta* (140721-10, holotype). **(A)** Basidiospores, **(B)** basidia and basidioles, **(C)** cystidia, **(D)** pilocystidia, **(E)** caulocystidia, and **(F)** hyphae from context. (Bars: **(A)** = 5 μm, **(B–F)** = 10 μm). Drawn by Hai-Jiao Li.

***Podoscypha subinvoluta*** Jing Si & Hai J. Li sp. nov. (Figures 5, 6).

MycoBank no.: MB847439.

*Diagnosis*—*Podoscypha subinvoluta*is characterized by its partly tomentose, white to cream, buff, cinnamon, yellowish brown to clay-buff pileal surface, absence of gloeocystidia, thick-walled, mostly lanceolate cystidia, presence of pilocystidia and caulocystidia, small, thin-walled, ellipsoid to broadly ellipsoid basidiospores; discovered on angiosperms from tropical China.

*Holotype*—China. Yunnan Province, Xishuangbanna, Mengla County, Xishuangbanna Tropical Botanical Garden, on fallen angiosperm trunks, 140721-10 (GenBank accession numbers: OQ305836-ITS, OQ305831-LSU).

*Etymology*—*subinvoluta* referring to similarity with *P. involuta*.

*Habitat and distribution*—Gregarious on fallen angiosperm trunks, at present only discovered from its type locality, summer.

*Known distribution*—China (Yunnan).

*Description*—*Basidiocarps* annual, gregarious, stipitate, without odor or taste, corky when fresh and hard corky upon drying. *Pilei* flabelliform or reniform, up to 4.5 cm high and 6.5 cm wide. *Pileal surface* tomentose near the stipe and glabrous near the margin, concentrically and radially zonate; most parts white to cream, buff, cinnamon, yellowish brown to clay-buff near the stipe when fresh, flesh-pink to clay-pink to cinnamon when dry. *Hymenophore surface* smooth, cream, buff, to cinnamon when fresh, pinkish buff to clay-buff when dry. *Stipe* surface glabrous, cinnamon to yellowish brown when fresh, color similar to the pileal surface when dry, up to 1.2 cm long and 1.5–3 mm in diameter.

*Hyphal structure*—dimitic, generative hyphae with clamps, hyaline, thin-to thick-walled, branched; skeletal hyphae colorless, thick-walled with a wide to narrow lumen, unbranched to rarely branched; IKI−, CB−, tissues unchanged in KOH. Generative hyphae in the pileal context common, hyaline, thin-to thick-walled, branched, 2–5 μm in diameter; skeletal hyphae in the pileal context dominant, hyaline, thick-walled, with a wide to narrow lumen, unbranched to rarely branched, more or less parallel along the pileal surface, 3–6 μm in diameter; generative hyphae in the stipe trama similar to that in the pileal context, 3–4 μm in diameter; skeletal hyphae in the stipe trama similar to that in the pileal context, 4–7 μm in diameter, branched skeletal hyphae rare, thick-walled to subsoil, 2–3.5 μm in diameter, may be easily misinterpreted as binding hyphae.

*Microstructure*—*Basidia* clavate to cylindrical, with 4 sterigmata and a basal clamp, 18–25 × 4–5 μm; basidioles dominant, shape similar to basidia, but slightly smaller; *Gloeocystidia* not observed; *Cystidia* abundant, hyaline, thick-walled, mostly lanceolate, sometimes obpyriform or subcylindrical, rarely with crystals on the apex, which dissolve rapidly in KOH, may arise at any level in the thickened hymenium or buried under successive layers of basidia, 30–50 × 7–12 μm; *Pilocystidia* hyaline, clavate to cylindrical, thick-walled, with secondary septata, 20–64 × 6–9 μm; *Caulocystidia* form a paliform cutis, hyaline, clavate to cylindrical, thick-walled, encrusted with irregular crystals and strongly glutinized, 36–60 × 4–8 μm; *Basidiospores* ellipsoid to broadly ellipsoid, rarely subglobose, hyaline, thin-walled, smooth, IKI−, CB−, [40/1/1] 2.5–3.7 (−3.8) × 2–2.7 (−2.8) μm, L = 3.02 μm, W = 2.31 μm, Q = (1.07−) 1.14–1.52 (−1.73), and Q_m_ = 1.31 ± 0.14.

***Podoscypha tropica*** Jing Si & Hai J. Li sp. nov. (Figures 5, 6).

MycoBank no.: MB847440.

**Figure 7:**
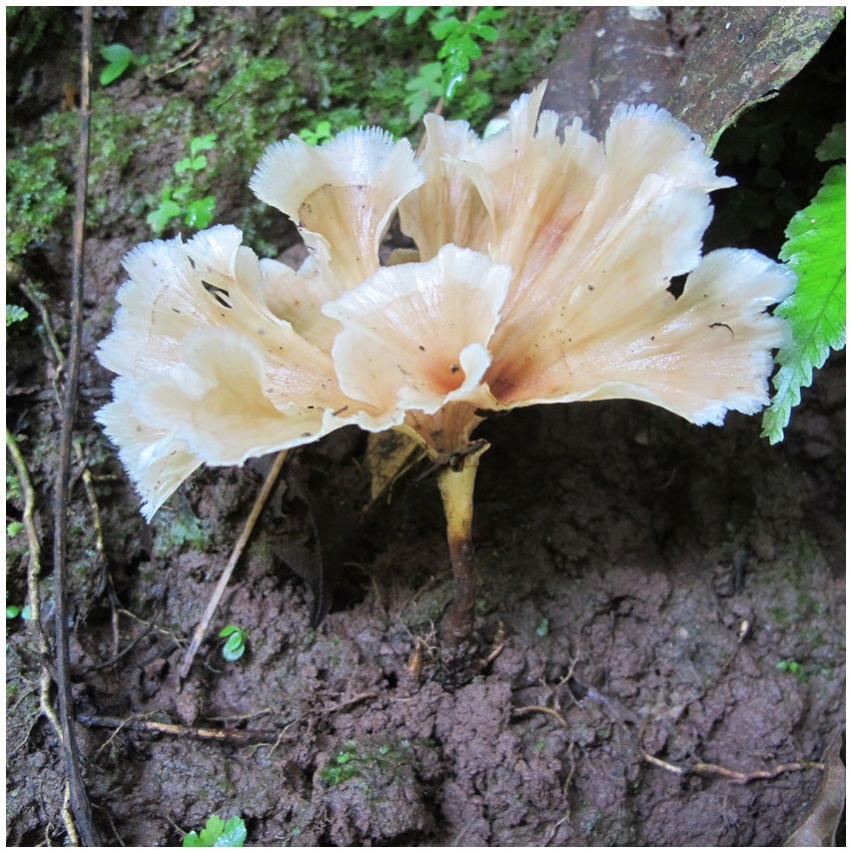
Basidiomata of *Podoscypha tropica*.

**Figure 8:**
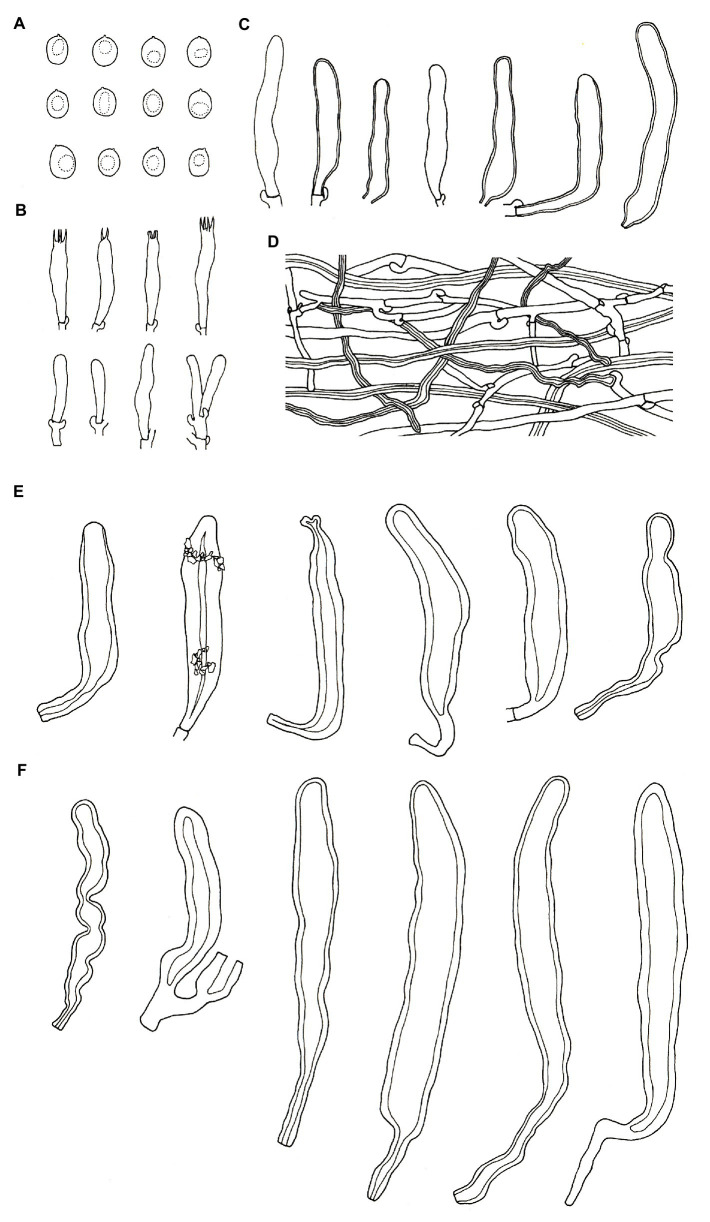
Microscopic structures of *Podoscypha tropica* (140719-19, holotype). **(A)** Basidiospores, **(B)** basidia and basidioles, **(C)** gloeocystidia, **(D)** hyphae from context, **(E)** pilocystidia, and **(F)** caulocystidia. (Bars: **(A)** = 5 μm, **(B–F)** = 10 μm). Drawn by Hai-Jiao Li.

*Diagnosis*—*Podoscypha tropica* is characterized by a glabrous, yellowish-brown, pinkish-buff to brownish-orange pileal surface with cream, buff to pinkish buff margin, presence of gloeocystidia, pilocystidia, and caulocystidia without secondary septata, small, thin-walled, ellipsoid to broadly ellipsoid basidiospores; discovered on angiosperms from tropical China (Figures 7, 8).

*Holotype*—China. Yunnan Province, Xishuangbanna, Mengla County, Wangtianshu Park, N: 21°37′27.81″E: 101°35′13.12″, on fallen angiosperm trunks, 140719-19 (GenBank accession numbers: OQ305834-ITS, OQ305830-LSU).

*Etymology*—*tropica* referring to the species being distributed in the tropics.

*Habitat and distribution*—Gregarious on fallen angiosperm trunks or fallen angiosperm branches, at present only discovered from its type locality, summer.

*Known distribution*—China (Yunnan).

*Description*—*Basidiocarps* annual, gregarious, stipitate, without odor or taste, corky when fresh, and hard corky upon drying. *Pilei* spathulate, reniform, flabellate to pseudoinfundibuliform, up to 5 cm high and 6 cm wide. *Pileal surface* glabrous, yellowish brown, pinkish buff to brownish orange at the center and near the stipe when fresh, clay-buff to orange-brown when dry. The margin cream, buff to pinkish buff when fresh, clay-buff to orange-brown when dry, and strongly dentate. *Hymenophore surface* smooth, cream to buff when fresh, cinnamon to clay-buff when dry. *Stipe* surface glabrous, buff to straw-yellow on the upper part, and dark brown to fuscous toward the base when fresh, and the color slightly paler when dry, up to 5 cm long and 2–4 mm in diameter.

*Hyphal structure*—dimitic, generative hyphae with clamps, hyaline, thin-walled, branched; skeletal hyphae colorless, thick-walled with a wide to narrow lumen, unbranched to rarely branched; IKI−, CB−, tissues unchanged in KOH. Generative hyphae in the pileal context common, hyaline, thin-walled, branched, interwoven, 2–4 μm in diameter; skeletal hyphae in the pileal context dominant, hyaline, thick-walled, with a wide to the narrow lumen, unbranched to rarely branched, more or less parallel along the pileal surface, 2–5 μm in diameter; generative hyphae in the stipe trama similar to that in the pileal context, 2–4.5 μm in diameter; skeletal hyphae in the stipe trama similar to that in the pileal context, 2–4 μm in diameter.

*Microstructure*—*Basidia* clavate to cylindrical, with 4 sterigmata and a basal clamp, 25–33 × 4–5.5 μm; basidioles dominant, in shape similar to basidia, but slightly smaller; *Gloeocystidia* common, thin-to thick-walled, cylindrical to subcylindrical, usually swollen near the base and with obtuse apex, 45–75 × 7–10 μm; *Cystidia* absent; *Pilocystidia* hyaline, clavate to cylindrical, thick-walled, without secondary septata, sometimes encrusted with irregular crystals, 50–150 × 10–18 μm; *Caulocystidia* more or less similar to pilocystidia, 71–177 × 6–21 μm; *Basidiospores* ellipsoid to broadly ellipsoid, hyaline, thin-walled, smooth, often monoguttulate, IKI−, CB−, [60/2/2] 4.2–6.1 (−6.7) × (3−) 3.3–4.6 (−5) μm, L = 5.28 μm, W = 3.97 μm, Q = (1.09−) 1.14–1.53 (−1.83), and Q_m_ = 1.34 ± 0.15.

*Additional specimen examined (paratype)*—China. Yunnan Province, Dehong, Ruili, Mengxiu Town, Nanjingli Village, Alt.: 1,247 m; N: 24°7′27″E: 97°49′28″, on fallen angiosperm branches, 20190729-69 (GenBank accession numbers: OQ305835-ITS, OQ305829-LSU).

## 4. Discussion

In total, three subspecies of *Podoscypha petalodes* have been described: *P. petalodes* subsp. *floriformis*, *P. petalodes* subsp. *petalodes*, and *P. petalodes* subsp. *rosulate*. All three have gloeocystidia, but without cystidia in the hymenium (Duss, 1904; Reid, 1965). The new subspecies, *P. petalodes* subsp. *cystidiata*, can be easily distinguished from the others by its fusoid, thin-to thick-walled cystidia.

*Podoscypha subinvoluta*is similar to *P. involuta* by its partly tomentose concentrically and radially zonate pileal surface, mostly lanceolate cystidia, pilocystidia clavate to cylindrical, thick-walled, and small basidiospores; however, *P. subinvoluta* lacks gloeocystidia but has caulocystidia and larger basidiospores (2.5–3.7 × 2–2.7 μm vs. 2–3 × 1.75–2 μm, Reid, 1965). The partial ITS1 sequence of *P. subinvoluta* was obtained. Among the 279 bp aligned sequences, it clustered with one sequence of *P. involuta* (JN649357) from Thailand (only 2 bp sites were different), whereas it differed from the other two *P. involuta* sequences (JN649358 and MH861804) by 23 bp sites. Thus, we speculate that the specimen of *P. involuta* (JN649357) may be *P. subinvoluta*. The LSU sequence from the type specimen of *P. subinvoluta* grouped with two *P. involuta* sequences, and only 5 bp sites were different in the 915 bp aligned sequences.

In the phylogenetic analyses, *P. tropica* clustered with *P. cristata*, *P. brasiliensis*, *P. bubalina*, *P. bolleana*, *P. disseminate*, etc. Compared to the new species, *P. cristata* is lacking in pilocystidia and caulocystidia and has distinctly smaller basidiospores (4–4.75 × 1.75–2 μm, Reid, 1965). *Podoscypha brasiliensis* differs from the new species in having a minute tomentose pileal surface, the absence of pilocystidia and caulocystidia, and slightly longer and narrower basidiospores (5–7 × 3.5–4 μm, Reid, 1965). *Podoscypha bubalina* differs from *P. tropica* by having truly infundibuliform basidiocarps, the absence of pilocystidia, and smaller basidiospores (3.75–4.75 × 2.5–3.2 μm, Reid, 1965). *Podoscypha bolleana* is similar to *P. tropica* in having gloeocystidia, pilocystidia, and caulocystidia, but can be easily differentiated by its smaller basidiospores (4–5 × 2.75–3.2 μm, Reid, 1965). Similar to *P. tropica*, *P. disseminata* also has gloeocystidia, pilocystidia, and caulocystidia; however, *P. disseminata* has pilocystidia and caulocystidia with secondary septata, chlamydospores in the context, and slightly narrower basidiospores (4.5–6 × 3–4 μm, Douanla-Meli and Langer, 2004).

*Podoscypha yunnanensis* was a new species described in Yunnan Province, China (Wu et al., 2019). It has only caulocystidia without gloeocystidia and pilocystidia, which can be easily distinguished from the two new species and one new subspecies discovered in this study. The diversity of *Podoscypha* in China is poorly understood, especially in the subtropical and tropical regions. Further studies are required to reveal the species diversity in China.

## Data availability statement

The datasets presented in this study can be found in online repositories. The names of the repository/repositories and accession number(s) can be found at: https://www.ncbi.nlm.nih.gov/genbank/, OQ305832–OQ305836; https://www.ncbi.nlm.nih.gov/genbank/, OQ305827–OQ305831.

## Author contributions

H-JL designed the research. H-JL, JS, Y-ZZ, and J-QL prepared the samples, conducted molecular experiments, and analyzed the data. H-JL and JS discussed the results and drafted and edited the manuscript. JS carried out the project administration and funding acquisition. All authors contributed to the manuscript and approved the submitted version.

## Funding

The research was financed by the National Natural Science Foundation of China (32270016 and 32070016).

## Conflict of interest

The authors declare that the research was conducted in the absence of any commercial or financial relationships that could be construed as a potential conflict of interest.

## Publisher’s note

All claims expressed in this article are solely those of the authors and do not necessarily represent those of their affiliated organizations, or those of the publisher, the editors and the reviewers. Any product that may be evaluated in this article, or claim that may be made by its manufacturer, is not guaranteed or endorsed by the publisher.

## References

[ref1] Bernicchia A. Gorjón S. P. (2010). Fungi Europaei 12: Corticiaceaes l. Edizioni Candusso, Lomazzo.

[ref2] Binder M. Justo A. Riley R. Salamov A. López-Girálde F. Sjökvist E.. (2013). Phylogenetic and phylogenomic overview of the Polyporales. Mycologia 105, 1350–1373. doi: 10.3852/13-003, PMID: 23935031

[ref3] Boidin J. (1959). Hétérobasidiomycèstes saprophytes et Homobasidiomycètesrésupinés: VI. Essai sur le genre *Stereum* sensulato. Rev. Mycol. 24, 197–225.

[ref4] Boidin J. (1960). Le genre *Stereum* Pers. S.L. au Congo belge. Bulletin du Jardin botanique de l'État a Bruxelles 30, 283–355. doi: 10.2307/3667306

[ref5] Chenna R. Sugawara H. Koike T. Lopez R. Gibson T. J. Higgins D. G.. (2003). Multiple sequence alignment with the Clustal series of programs. Nucleic Acids Res. 31, 3497–3500. doi: 10.1093/nar/gkg500, PMID: 12824352 PMC168907

[ref6] Crozier J. Thomas S. E. Aime M. C. Evans H. C. Holmes K. A. (2006). Molecular characterization of fungal endophytic morphospecies isolated from stems and pods of *Theobroma cacao*. Plant Pathol. 55, 783–791. doi: 10.1111/j.1365-3059.2006.01446.x

[ref7] Dai Y. C. (2011). A revised checklist of corticioid and hydnoid fungi in China for 2010. Mycoscience 52, 69–79. doi: 10.1007/s10267-010-0068-1

[ref8] Dhingra G. S. (1987). The genus *Phlebiopsis* in the eastern Himalayas. Nova Hedwig. 44, 221–227.

[ref9] Douanla-Meli C. Langer E. (2004). A taxonomic study of the family Podoscyphaceae (Basidiomycetes), new species and new records in Cameroon. Mycotaxon 90, 323–335.

[ref10] Drechsler-Santos E. R. Gibertoni T. B. de Queiroz Cavalcanti M. A. (2007). *Podoscypha aculeata*, a new record for the neotropics. Mycotaxon 101, 69–72.

[ref11] Duss R. P. (1904). *Flore Cryptogamique des Antilles Françaises*. Impr. et Lith. Lucien Declume.

[ref12] Hall T. A. (1999). Bio edit: a user-friendly biological sequence alignment editor and analysis program for windows 95/98/NT. Nucleic Acids Symp. Ser. 41, 95–98.

[ref13] Hibbett D. S. Binder M. (2002). Evolution of complex fruiting-body morphologies in homobasidiomycetes. Proc. R. Soc. Lond. B Biol. Sci. 269, 1963–1969. doi: 10.1098/rspb.2002.2123, PMID: 12396494 PMC1691125

[ref14] Huelsenbeck J. P. Crandall K. A. (1997). Phylogeny estimation and hypothesis testing using maximum likelihood. Annu. Rev. Ecol. Syst. 28, 437–466. doi: 10.1146/annurev.ecolsys.28.1.437

[ref15] Justo A. Miettinen O. Floudas D. Ortiz-Santana B. Sjökvist E. Lindner D.. (2017). A revised family-level classification of the Polyporales (Basidiomycota). Fungal Biol. 121, 798–824. doi: 10.1016/j.funbio.2017.05.010, PMID: 28800851

[ref16] Larsson K. H. (2007). Re-thinking the classification of corticioid fungi. Mycol. Res. 111, 1040–1063. doi: 10.1016/j.mycres.2007.08.001, PMID: 17981020

[ref17] Li H. J. Xie J. W. Zhang S. Zhou Y. J. Ma P. B. Zhou J.. (2015). *Amanita subpallidorosea*, a new lethal fungus from China. Mycol. Prog. 14:43. doi: 10.1007/s11557-015-1055-x

[ref18] Miettinen O. Larsson E. Sjökvist E. Larsson K. H. (2012). Comprehensive taxon sampling reveals unaccounted diversity and morphological plasticity in a group of dimitic polypores (Polyporales, Basidiomycota). Cladistics 28, 251–270. doi: 10.1111/j.1096-0031.2011.00380.x, PMID: 34872189

[ref19] Moncalvo J. M. Vilgalys R. Redhead S. A. Johnson J. E. James T. Y. Aime M. C.. (2002). One hundred and seventeen clades of euagarics. Mol. Phylogenet. Evol. 23, 357–400. doi: 10.1016/S1055-7903(02)00027-1, PMID: 12099793

[ref20] Nylander J. A. A. (2004). MrModeltest V2. Program Distributed by the Author. Uppsala: Evolutionary Biology Centre, Uppsala University.

[ref21] Page R. D. (1996). Tree view: an application to display phylogenetic trees on personal computers. Comput. Appl. Biosci. 12, 357–358. doi: 10.1093/bioinformatics/12.4.357 8902363

[ref22] Patouillard N. T. (1900). Essaitaxonomique sur les Familles et les Genres des Hyménomycètes. Lucien Declume Press, Lons-Le-Saunier.

[ref23] Petersen J. H. (1996). The Danish Mycological Society's Colour-Chart. Foreningentil Svampekundskabens Fremme, Greve, Denmark.

[ref24] Posada D. Crandall K. A. (1998). MODELTEST: testing the model of DNA substitution. Bioinformatics 14, 817–818. doi: 10.1093/bioinformatics/14.9.817, PMID: 9918953

[ref25] Posada D. Crandall K. A. (2001). Selecting the best-fit model of nucleotide substitution. Syst. Biol. 50, 580–601. doi: 10.1080/106351501750435121, PMID: 12116655

[ref26] Reid D. A. (1965). A monograph of the stipitate stereoid fungi. Nova Hedwig. Beih. 18, 1–382.

[ref27] Ronquist F. Huelsenbeck J. P. (2003). MrBayes 3: Bayesian phylogenetic inference under mixed models. Bioinformatics 19, 1572–1574. doi: 10.1093/bioinformatics/btg180, PMID: 12912839

[ref28] Ryvarden L. (1997). Podoscypha warneckii. Mycotaxon 64, 401–403.

[ref29] Ryvarden L. (2015). Type studies in *Stereums*. Lato 5. Species described by M.J. Berkeley. Synopsis Fungorum 33, 13–19.

[ref30] Ryvarden L. (2020). The genus *Stereum*− a synopsis. Synopsis Fungorum 40, 46–96.

[ref31] Sjökvist E. Larsson E. Eberhardt U. Ryvarden L. Larsson K. H. (2012). Stipitate stereoid basidiocarps have evolved multiple times. Mycologia 104, 1046–1055. doi: 10.3852/11-174, PMID: 22492407

[ref32] Stamatakis A. (2006). RAxML-VI-HPC: maximum likelihood-based phylogenetic analyses with thousands of taxa and mixed models. Bioinformatics 22, 2688–2690. doi: 10.1093/bioinformatics/btl446, PMID: 16928733

[ref33] Swofford D. L. (2002). PAUP: Phylogenetic Analysis Using Parsimony, Version 4.0 Beta 10. Sinauer Associates, Sunderland.

[ref34] Vilgalys R. Hester M. (1990). Rapid genetic identification and mapping of enzymatically amplified ribosomal DNA from several *Cryptococcus* species. J. Bacteriol. 172, 4238–4246. doi: 10.1128/jb.172.8.4238-4246.1990, PMID: 2376561 PMC213247

[ref35] Vu D. Groenewald M. de Vries M. Gehrmann T. Stielow B. Eberhardt U.. (2019). Large-scale generation and analysis of filamentous fungal DNA barcodes boosts coverage for kingdom fungi and reveals thresholds for fungal species and higher taxon delimitation. Stud. Mycol. 92, 135–154. doi: 10.1016/j.simyco.2018.05.001, PMID: 29955203 PMC6020082

[ref36] White T. J. Bruns T. Lee S. Taylor J. (1990). “Amplification and direct sequencing of fungal ribosomal RNA genes for phylogenetics” in PCR Protocols: A Guide to Methods and Applications. eds. Innis M. A. Gelfand D. H. Sninsky J. J. White T. J. (New York: Academic Press)

[ref37] Wu S. H. (2003). Lignicolous homobasidiomycetes newly recorded from Taiwan. Mycotaxon 88, 373–376.

[ref38] Wu Y. X. Shen S. Zhao C. L. (2019). *Podoscypha yunnanensis* sp. nov. (Polyporales, Basidiomycota) evidenced by morphological characters and phylogenetic analyses. Phytotaxa 387, 210–218. doi: 10.11646/phytotaxa.387.3.2

